# Investigating the Factors Contributing to the Lower Prevalence of Hyperlipidemia in Hispanic or Latinx Adults Compared to Non-Hispanic White Adults

**DOI:** 10.7759/cureus.88386

**Published:** 2025-07-20

**Authors:** Valentina Roa Forster, Alejandra Viera Plasencia, Lisett Castellanos, Eneida Roldan

**Affiliations:** 1 Epidemiology and Biostatistics, Florida International University, Herbert Wertheim College of Medicine, Miami, USA; 2 Hematology and Oncology, Florida International University, Herbert Wertheim College of Medicine, Miami, USA; 3 Pathology, Florida International University, Herbert Wertheim College of Medicine, Miami, USA

**Keywords:** cardiovascular risk, healthcare access, health disparities, hispanic health, hyperlipidemia, latinx population, national health interview survey, preventive care, social determinants of health, underdiagnosis

## Abstract

Background

Hyperlipidemia is a major risk factor for cardiovascular disease, yet Hispanic/Latinx adults in the United States report lower prevalence compared to non-Hispanic White adults despite similar or greater cardiovascular risk factors.

Objective

To examine disparities in reported hyperlipidemia among Hispanic/Latinx adults and assess the role of social determinants of health, healthcare access, and cultural factors.

Methods

A cross-sectional, descriptive analysis was conducted using publicly available summary-level data from the National Health Interview Survey (NHIS) from 2019 to 2022. Weighted prevalence estimates and 95% confidence intervals were compared across ethnic groups. Differences between groups were considered statistically significant if their 95% confidence intervals did not overlap, in accordance with standard practice for analyzing summary-level survey data.

Results

Hispanic/Latinx adults had significantly lower rates of reported hyperlipidemia (16.4%) and angina (1.1%) compared to non-Hispanic White adults (23.3% and 1.8%, respectively). They also reported fewer wellness visits, lower prescription medication use, and higher rates of uninsurance. These disparities suggest potential underdiagnosis rather than a true difference in disease burden.

Conclusion

The findings underscore the impact of structural inequities, limited healthcare access, and language barriers on chronic disease diagnosis in Hispanic/Latinx populations. Differences were considered statistically significant based on non-overlapping 95% confidence intervals. As this study relied on self-reported, summary-level data, potential reporting bias and inability to adjust for confounders should be considered. Culturally tailored interventions, bilingual care, and community health worker outreach may help close these gaps and promote equitable cardiovascular outcomes.

## Introduction

Hyperlipidemia, a disorder defined by a total serum cholesterol level ≥200 mg/dL, is a significant risk factor for cardiovascular diseases, including atherosclerosis, non-ischemic heart failure, and arrhythmias [1]. Studies show that it affects 30% of US adults. It is documented as an underlying cause of death in more than 60,000 adults between 2018 and 2022 [2]. On the other hand, social determinants of health (SDOH), such as access to healthcare, quality of healthcare, socioeconomic status, access to preventive and routine care, family and marital support, and educational levels, play a critical role in health outcomes and influence the impact and prevalence of hyperlipidemia [3,4].

Hispanics/Latinx, the largest minority group in the United States, comprise more than 15% of the US population and are defined by Latin American, Caribbean, and Spanish descent. As a group, they face significant health disparities exacerbated by social determinants of health (SDOH), including higher rates of uninsurance, limited access to preventive care, lower use of prescription medications, and language barriers that may contribute to underdiagnosis [5]. Although hyperlipidemia and its comorbidities have been widely studied in the general population, few investigations have focused on contributing factors specific to Hispanic or Latinx adults such as health beliefs, dietary practices, language barriers, and medical mistrust, which may shape healthcare-seeking behavior and underdiagnosis in this group. Remarkably, based on self-reported data, Hispanic/Latinx adults aged 18 years or older report a lower prevalence of hyperlipidemia compared to non-Hispanic White adults [6]. This discrepancy suggests a potential underreporting or difference in diagnosis of hyperlipidemia within Hispanic/Latinx in the United States when compared to their White counterparts.

Given the morbidity of angina and its correlation with cardiovascular events, prompt and adequate management of hyperlipidemia is crucial [7]. Understanding the prevalence of hyperlipidemia and its impact on angina can aid in timely diagnosis and treatment. This study seeks to explore the factors that contribute to the underreporting of hyperlipidemia in US Hispanics/Latinx adults by considering the impact of SDOH, cultural health beliefs, and practices characteristic of this group. Furthermore, we aim to advance the understanding of barriers to accurate diagnosis of hyperlipidemia in Hispanics/Latinx and contribute to improved health outcomes and decreased disparities.

## Materials and methods

Study design

This study employed a cross-sectional, descriptive design, utilizing publicly available summary estimates from the National Health Interview Survey (NHIS) for the years 2019-2022.

Data source

The NHIS is a nationally representative survey of the non-institutionalized US population conducted by the Centers for Disease Control and Prevention (CDC). It collects data through household interviews using a geographically clustered sampling method, capturing approximately 27,000 adult respondents each year from all 50 states and the District of Columbia. Following the COVID-19 pandemic, the survey methodology transitioned from in-person to telephone-based interviews. This shift may have influenced response patterns, particularly among populations with limited English proficiency. The NHIS collects information on sociodemographic characteristics, health conditions, behaviors, and healthcare access and utilization.

Study population

This analysis included adults aged 18 years and older identified as either Hispanic or Latinx or non-Hispanic White, single race, based on NHIS summary tables. The estimates represent population-level averages derived from combined NHIS samples collected between 2019 and 2022. Other racial and ethnic groups were not included in this analysis due to the limited disaggregation available in the publicly accessible summary tables and to maintain a focused comparison aligned with the study’s objective.

Variables

The primary exposure variable in this study was ethnicity, specifically comparing adults aged 18 years and older who identified as Hispanic or Latinx adults (HA) versus those who identified as non-Hispanic White, single-race adults (NHWA). The outcome variables included a range of self-reported health indicators and healthcare utilization measures, as reported in the NHIS summary tables. These outcomes were: high cholesterol (primary outcome), angina pectoris, wellness visits, prescription medication use, fair or poor self-rated health, hospital emergency department visits, being uninsured for more than one year, frequent experiences of worry, nervousness, or anxiety, and receipt of counseling from a mental health professional. NHIS defines “high cholesterol” as the self-reported physician diagnosis of high blood cholesterol. “Wellness visits” refer to routine general checkups within the past 12 months. All variables were reported as weighted percentages with corresponding 95% confidence intervals.

Statistical analysis

Due to the use of publicly available, aggregated summary data from the NHIS, this study employed a descriptive analytic approach. Summary-level estimates were retrieved from the NHIS interactive data query system for each year from 2019 to 2022. Weighted percentages and 95% confidence intervals were extracted manually from the online tables; no inferential statistical software was used, given the descriptive nature of the analysis. Weighted prevalence estimates with 95% confidence intervals were extracted for each outcome variable, stratified by ethnicity (Hispanic/Latinx vs. non-Hispanic White, single race). All estimates were derived from NHIS summary tables, which include pre-applied sampling weights and exclude respondents with missing data, as per CDC methodology. Between-group comparisons were assessed by evaluating point estimate differences and the degree of overlap between confidence intervals. In accordance with the established practice for analyzing summary-level survey data, non-overlapping confidence intervals were interpreted as evidence of statistically meaningful differences. While individual-level statistical tests such as chi-square or t-tests could not be conducted, variables demonstrating notable disparities between groups were identified as potential confounders. These included insurance status, prescription medication use, and engagement in preventive care. Although multivariable regression modeling was not feasible with summary-level data, future analyses using NHIS microdata are warranted to quantify adjusted associations between ethnicity and reported high cholesterol prevalence, accounting for these factors, which could allow for adjustment of confounders and strengthen causal inferences.

P-values could not be calculated due to the use of publicly available, summary-level NHIS data, which do not include raw, respondent-level information necessary for formal hypothesis testing. The NHIS summary tables are designed for descriptive analyses and do not provide standard errors or design variables needed for complex survey-based inferential statistics. As such, statistical significance was interpreted conservatively using non-overlapping 95% confidence intervals, a common and accepted approach for comparative analyses of summary data in NHIS reports.

## Results

Between 2019 and 2022, HA consistently reported lower rates of high cholesterol (16.4%; 95% CI: 15.0%-17.7%) compared to NHWA (23.3%; 95% CI: 22.7%-24.1%). However, a similar pattern was observed between the two groups for angina pectoris, with HA reporting a prevalence of 1.1% (95% CI: 0.8%-1.6%) versus 1.8% among NHWA (95% CI: 1.6%-2.0%). In both cases, the confidence intervals did not overlap, indicating statistically meaningful differences and suggesting lower reporting of cardiovascular disease among HA (Table 1).

**Table 1:** Comparison of health outcomes and healthcare access between Hispanic/Latinx and non-Hispanic White adults (2019–2022) P-values could not be calculated due to the use of publicly available, summary-level data, which lack the individual-level variance estimates required for inferential testing. Between-group differences were interpreted based on non-overlapping 95% confidence intervals.

	2019-2022 Averages	
	Percentage (%)	95% Confidence Interval
	High cholesterol for adults 18 years old and older (main outcome)	
Hispanic or Latinx	16.4%	(15.0%-17.7%)
Not Hispanic or Latinx, White, single race	23.3%	(22.7%-24.1%)
	Angina pectoris for adults 18 years old and older	
Hispanic or Latinx	1.1%	(0.8%-1.6%)
Not Hispanic or Latinx, White, single race	1.8%	(1.6%-2.0%)
	Wellness visit for adults 18 years old and older	
Hispanic or Latinx	71.5%	(69.8%-73.3%)
Not Hispanic or Latinx, White, single race	78.6%	(77.8%-79.4%)
	Hospital emergency department visit for adults 18 years old and older	
Hispanic or Latinx	19.8%	(18.3%-21.4%)
Not Hispanic or Latinx, White, single race	18.7%	(18.0%-19.4%)
	Prescription medication use for adults 18 years old and older	
Hispanic or Latinx	51.2%	(49.3%-53.4%)
Not Hispanic or Latinx, White, single race	72.1%	(71.3%-73.0%)
	Fair or poor health status for adults 18 years old and older	
Hispanic or Latinx	16.2%	(14.8%-17.8%)
Not Hispanic or Latinx, White, single race	13.2%	(12.6%-13.9%)
	Uninsured for more than one year for adults 18 years old and older	
Hispanic or Latinx	21.9%	(20.0%-23.9%)
Not Hispanic or Latinx, White, single race	5.3%	(4.8%-5.8%)
	Regularly had feelings of worry, nervousness, or anxiety for adults 18 years old and older	
Hispanic or Latinx	9.4%	(8.3%-10.5%)
Not Hispanic or Latinx, White, single race	12.9%	(12.3%-13.5%)
	Counseled by a mental health professional for adults 18 years old and older	
Hispanic or Latinx	7.9%	(7.0%-9.1%)
Not Hispanic or Latinx, White, single race	12.2%	(11.6%-12.8%)

HA also experienced notable disparities in healthcare access and utilization. Wellness visit rates were lower among HA (71.5%; 95% CI: 69.8%-73.3%) than NHWA (78.6%; 95% CI: 77.8%-79.4%), as were rates of prescription medication use (51.2%; 95% CI: 49.3%-53.4%) in HA vs. (72.1%; 95% CI: 71.3%-73.0%) in NHWA). Additionally, 21.9% of HA (95% CI: 20.0%-23.9%) reported being uninsured for more than one year, compared to just 5.3% of NHWA (95% CI: 4.8%-5.8%). These differences were all statistically significant.

Overall self-rated health was also worse among HA, with 16.2% (95% CI: 14.8%-17.8%) reporting fair or poor health, compared to 13.2% of NHWA (95% CI: 12.6%-13.9%). In contrast, emergency department visit rates were similar between groups: 19.8% in HA (95% CI: 18.3%-21.4%) and 18.7% in NHWA (95% CI: 18.0%-19.4%), with overlapping confidence intervals possibly indicating no significant difference. This similarity may reflect greater reliance on emergency departments among HA for acute care needs due to barriers in accessing preventive or routine outpatient services.

Taken together, statistically significant differences were observed in all outcomes except emergency department use. These findings suggest that lower reported rates of hyperlipidemia and angina among HA may reflect disparities in healthcare access and underdiagnosis rather than actual differences in cardiovascular disease burden. P-values could not be reported due to the use of publicly available, summary-level NHIS data, which do not include individual-level variance estimates or design variables required for inferential testing, as outlined in CDC methodology.

## Discussion

This analysis of NHIS data from 2019 to 2022 reveals a consistent disparity in the reporting of hyperlipidemia between HA and NHWA. The Hispanic/Latinx population is highly heterogeneous, with health outcomes and healthcare access varying by country of origin, nativity, and acculturation level, which are factors not captured in the summary-level data but critical for future disaggregated analyses. Despite HA demonstrating higher rates of risk indicators, such as fair or poor self-rated health and similar rates of angina pectoris and emergency department (ED) utilization, their reported prevalence of hyperlipidemia was substantially lower. This discrepancy, observed alongside comparable cardiovascular outcomes, suggests a potential underdiagnosis of hyperlipidemia in the HA population. These factors, including language barriers, health literacy, and acculturation, were not directly measured but are interpreted based on existing literature and population-level disparities observed in NHIS summary data.

Although healthcare spending has steadily increased throughout the years, a study following a minority group from 1999 to 2018 in the United States, including Hispanic/Latinx individuals, showed persistent health inequities despite this increased investment [8]. The lack of healthcare access among HA compared to NHWA is well-documented. Hispanic/Latinx adults face disadvantages in education, employment, income, and health insurance - factors that directly impact their ability to obtain preventive care and chronic disease management. Those with limited English proficiency are especially unlikely to have a usual source of care or to utilize healthcare services [9].

Language and communication barriers further compound these disparities. Difficulty in understanding medical advice may lead to reduced adherence, lower satisfaction with care, and increased risk of misdiagnosis or delayed diagnosis. Interestingly, even among Hispanics/Latinx who report comfort with English, disparities persist: they are three times more likely to be uninsured and twice as likely to have lower family income compared to NHWA, suggesting that systemic inequities extend beyond language alone [10].

Other factors that can be taken into consideration regarding Hispanic/Latinx’s attitude towards preventative care are time from migration and acculturation to the United States. Acculturation is defined as “immigrant adoption of the behaviors and attitudes of the mainstream culture” [11]. Language proficiency and preference have been identified as indicators of Hispanic/Latinx immigrants’ acclimation to US culture. While some studies suggest that higher acculturation is associated with increased access to healthcare and greater utilization of preventive services, others indicate a decline in certain health behaviors with increased acculturation. For example, more acculturated Hispanic/Latinx adults are more likely to receive cholesterol screenings compared to those with limited English proficiency or recent immigration status. These patterns suggest that acculturation plays a nuanced role in shaping screening behavior and access to care, including hyperlipidemia diagnosis. In conjunction, these factors might be contributing to HA not seeking preventive care as well as not receiving proper primary care [10-13].

Although this study compares Hispanic/Latinx adults as a single group, it is important to acknowledge the substantial heterogeneity within this population. Health outcomes and healthcare access differ significantly by national origin, nativity (US-born vs. foreign-born), and level of acculturation. For example, Puerto Rican adults tend to report higher rates of chronic illness, while Mexican and Central American immigrants often face greater barriers to care, including limited English proficiency, documentation status, and lower insurance coverage [11]. Nativity also influences access to care and health behaviors. US-born Hispanic/Latinx adults are generally more likely to engage with the healthcare system, yet may adopt less healthy dietary and lifestyle habits than their foreign-born counterparts [13]. Acculturation, including duration of residence in the United States and language use, further modifies health risks and healthcare utilization, sometimes in conflicting ways [14,15]. Future research should disaggregate data by country of origin, nativity, and acculturation to inform more targeted interventions and improve outcomes across diverse Hispanic/Latinx communities.

Studies have shown that Hispanics/Latinx are underutilizing healthcare, which places them at an increased risk of lack of appropriate screenings, including diabetes, hypercholesterolemia, and cancer. In addition to healthcare access factors, potential differences in dietary patterns or genetic predispositions may also contribute to variation in hyperlipidemia prevalence, though these influences remain less well studied in Hispanic/Latinx populations [16]. A key factor playing a role in underdiagnosis is lack of insurance [11]. Several other factors contribute to the low utilization of healthcare by HA. The most frequently cited challenge is limited English proficiency, as the healthcare system was designed around English-speaking patients and they have trouble navigating it. This barrier causes confusion, frustration, and perception of poor care. Cultural traditions also impact patient experience in US healthcare. A study that conducted a survey of 499 Latinx patients in rural Oregon identified perceived discrimination and medical mistrust to impact healthcare utilization among this population. They agree with statements such as “my ethnic group cannot trust doctors.” Hispanics/Latinx have also been found to agree with fatalistic statements expressing that if bad things happen, they are meant to be [14,17,18].

Health literacy is lower among HA in the United States compared to NHWA, with the highest prevalence of low health literacy observed among older adults and those with limited English proficiency. The American Heart Association recognizes that low health literacy is a key driver of disparities in chronic disease outcomes, therapy adherence, and healthcare utilization, particularly in cardiovascular disease, diabetes, and obesity [19]. On the other hand, structural racism affects Hispanic/Latinx populations in the United States through exclusionary health insurance, underinvestment in Hispanic/Latinx neighborhoods, and persistent language barriers. This results in higher rates of uninsurance, reduced access to primary care, and lower healthcare accessibility for Latinx populations [20]. Digital divide is another key consideration in health disparities. This is defined as a gap in access to and effective use of digital health technologies. HA that prefer Spanish or have limited English proficiency have lower rates of internet access, less confidence using digital health tools, and greater difficulty evaluating and applying online health information. After the COVID-19 pandemic, telehealth services have become significantly prominent. These new technologies and challenges further limit engagement with patient portals and telehealth services in the Hispanic/Latinx community [21,22].

It is also important to recognize that the Hispanic/Latinx population is not monolithic. Substantial variation exists across subgroups defined by country of origin, nativity, and acculturation level. For example, prior research has shown that Puerto Rican adults tend to report higher rates of chronic conditions such as diabetes and hypertension compared to Mexican or Central American groups, while access to healthcare may vary significantly based on immigration status and regional concentration. Nativity also plays a role, with US-born Hispanic/Latinx adults generally having better healthcare access but also higher prevalence of some cardiovascular risk factors. These differences may influence the likelihood of screening, diagnosis, and reporting of hyperlipidemia. Future analyses using individual-level data should explore how such subgroup differences shape disparities in chronic disease outcomes [23,24].

Addressing these inequities requires a multifaceted approach that targets structural, cultural, and systemic barriers within the healthcare system. To address the language barrier and cultural differences, the system must strive to expand bilingual providers, interpreters, and culturally tailored education materials, especially during wellness visits. This can facilitate accurate, trust-building communication, which is essential for shared decision-making, therapy adherence, and patient engagement. The American Heart Association states that linguistically appropriate and culturally competent care reduces disparities in cardiovascular outcomes and improves medication adherence among underrepresented groups, including HA [25]. Implementing community health worker (CHW) programs such as Promotores de Salud throughout the United States is one way to address this cultural barrier. Promotores and Promotoras de Salud are trusted lay members of the Hispanic/Latinx communities that function as CHW to deliver culturally and linguistically tailored health education, conduct screenings, and assist with care navigation. Evidence shows that this approach demonstrates feasibility, sustainability, and significant improvements in both clinical and behavioral cardiovascular risk factors [8,26,27].

This study’s primary strength lies in its use of recent, nationally representative data from the NHIS, allowing for generalizable insights into disparities in cardiovascular health and healthcare access among Hispanic/Latinx and non-Hispanic White adults in the United States. By focusing on multiple years of data (2019-2022), the analysis reflects post-pandemic shifts in care utilization. However, the use of summary-level, self-reported data limits the ability to control for confounders such as age, sex, socioeconomic status, and comorbidities, and may introduce recall or reporting bias. The cross-sectional design further prevents causal inference. Subgroup analyses by age or sex were not conducted and represent an important area for future research. Additionally, the NHIS transitioned from in-person to telephone-based interviews following the onset of the COVID-19 pandemic, which may have influenced response patterns, particularly among Hispanic/Latinx individuals with limited English proficiency or variable phone access. These methodological changes could affect the consistency and comparability of self-reported data across years. Finally, the study does not account for heterogeneity within the Hispanic/Latinx population, including differences by country of origin, nativity, or language proficiency, which may influence healthcare utilization and outcomes.

## Conclusions

In summary, this study highlights a lower reported prevalence of hyperlipidemia among Hispanic/Latinx adults despite comparable or greater cardiovascular risk indicators. This suggests potential underdiagnosis driven by barriers such as limited healthcare access, language discordance, and structural inequities. Group differences were considered statistically significant when 95% confidence intervals did not overlap, a standard approach when working with summary-level survey data. However, reliance on self-reported data introduces limitations, including recall and reporting bias.

To address these disparities, expanding access to bilingual care in primary care settings through the recruitment of multilingual providers, the use of medical interpreters, and culturally competent communication training can improve the diagnosis and treatment of chronic conditions. Promotores de Salud programs, which engage trusted community health workers to deliver health education, facilitate screenings, and support care navigation, represent a scalable and sustainable model for reaching underserved Hispanic/Latinx populations. Future research using individual-level data is essential to confirm these findings, assess subgroup differences, and inform targeted interventions that promote equity in cardiovascular care. Additionally, future studies should also examine the potential contributions of genetic predisposition and dietary patterns to hyperlipidemia risk in Hispanic/Latinx populations to complement the focus on social determinants of health. As this is a descriptive study based on summary-level data, the findings should be interpreted as exploratory and hypothesis-generating rather than conclusive, and future research using individual-level data is essential to adjust for key confounders and validate these associations.
